# Epithelial processed *Mycobacterium avium* subsp. *paratuberculosis* induced prolonged Th17 response and suppression of phagocytic maturation in bovine peripheral blood mononuclear cells

**DOI:** 10.1038/s41598-020-78113-8

**Published:** 2020-12-03

**Authors:** Hong-Tae Park, Hyun-Eui Park, Soojin Shim, Suji Kim, Min-Kyoung Shin, Han Sang Yoo

**Affiliations:** 1 Department of Infectious Disease, College of Veterinary Medicine, Seoul National University, Seoul, 08826 South Korea; 2 Department of Microbiology, Research Institute of Life Science, College of Medicine, Gyeongsang National University, Jinju, 52727 South Korea; 3 BioMax/N-Bio Institute, Seoul National University, Seoul, 08826 South Korea

**Keywords:** Microbiology, Pathogenesis

## Abstract

Johne’s disease (JD) caused by *Mycobacterium avium* subsp. *paratuberculosis* (MAP) is a chronic, wasting infectious disease in ruminants that causes enormous economic losses to the dairy and beef cattle industries. Understanding the mechanism of persistency of MAP is key to produce novel ideas for the development of new diagnostic methods or prevention techniques. We sought interactions between the host and MAP using epithelial passage model, which mimic initial stage of infection. From the transcriptomic analysis of bovine immune cells (PBMCs), it was suggested that infection through the epithelial cells elicited prolonged Th17-derived immune response, as indicated by upregulation of IL-17A, IL-17F and RORC until 120 h p.i., compared to directly infected PBMCs. Global downregulation of gene expression was observed after 72 h p.i., especially for genes encoding cell surface receptors of phagocytic cells, such as Toll-like receptors and MHC class II molecules. In addition, the cholesterol efflux transporters ABCA1, ABCG1, and APOE, which are regulated by the LXR/RXR pathway, were downregulated. In summary, it would be suggested that the host initiate immune response to activate Th17-derived cytokines, and MAP survives persistently by altering the host adaptive immune response by suppressing surface receptors and manipulating lipid metabolism in phagocytic cells.

## Introduction

*Mycobacterium avium* subsp. *paratuberculosis* (MAP) is a causative agent of Johne’s disease (JD), which causes granulomatous enteropathy in ruminants. The main symptom of the disease is a weakness due to chronic diarrhea and is accompanied by a decrease in milk production; thus, infected farms are economically damaging^1,2^. It is known that cows infected with MAP have an incubation period of more than 2 years until clinical symptoms develop^3^. During this period, infected cows excrete feces containing MAP without any clinical signs of the disease^3^. To detect the infected animals, serum ELISA and fecal PCR have been widely used. However, the detection sensitivity of these methods is low because fecal shedding occurs in an intermittent manner, and antibody levels against MAP in serum rise during a relatively later stage of infection^4,5^. This diagnostic difficulty is due to the nature of MAP infection. It is associated comprehensively with persistency of MAP that can evade host immune mechanisms at initial stage of infection and reactivation of MAP according to changes of immune system in infected animals, especially the immunological shift from Th1-related response to Th2-related response^6,7^. Despite the efforts of many researchers, the infectious nature (life cycle) of MAP has not been elucidated in detail at the molecular level. Therefore, identification of host-MAP interaction at the molecular level can provide a basis for discovering new genes for diagnosis or development of prevention techniques.

At the initial stage of infection, MAP enters the host intestine via M cells or enterocytes and is ingested by host macrophages or dendritic cells (DCs) in the lamina propria^5^. Like other mycobacteria, MAP can survive and replicate in non-activated phagocytes by inhibiting phagosome-lysosome maturation^8,9^. MAP eventually causes cell death, and released MAP is then phagocytosed by freshly accumulated macrophages and DCs activated by tumor necrosis factor α (TNF-α) and interferon γ (IFN-γ). Activated macrophages and DCs produce cytokines and chemokines, such as interleukin (IL)-1, IL-12, IL-18, C–C motif chemokine ligand 2 (CCL2), and C-X-C motif chemokine 10 (CXCL10), which recruit CD4+ T cells, CD8+ T cells, monocytes, neutrophils, and natural killer (NK) cells^10^. These recruited immune cells induce cell infiltration to the infection site and drive granuloma formation^11^. In the granuloma, MAP goes into a dormant state and persistently infects the host animal until it is reactivated by unknown immunological changes. In the later stages of infection, antibodies against MAP are frequently observed with bacterial shedding, which indicates disease progression^12^. It has been accepted that the shift in the immune response from classical Th1 to Th2 causes disease progression^13–15^. However, these Th1-Th2 shifts cannot accurately describe the immune responses in the host in relation to MAP persistence^16^. Rather, Th17-derived immune responses to MAP infection in the early stage have been the focus of many recent studies^17–19^. In our previous study, a novel model related to host response in subclinical infection was suggested in which downregulation of IL-17A, IL-17F, and IL-26, upregulation of PIP5K1C and loss of granuloma integrity resulted in fecal shedding and dissemination of pathogen^20^. Recently, γδ T cells have been implicated in the development and maintenance of granuloma induced by pathogenic mycobacteria^21^.

Thanks to recent advances in sequencing technology, RNA-seq based transcriptomic analyses with MAP infection have been performed in vitro and in vivo environments^22–25^. However, in vivo studies could not elucidate the initial stage of infection related to persistence, such as the immune response to the formation of granuloma, and in vitro studies could not represent the actual course of infection because the infection models were mainly based on single cell types, such as monocyte-derived macrophages (MDMs). In an actual infection situation, the pathogen is not directly exposed to immune cells, but after passing through the epithelial barrier, infection occurs in immune cells, such as macrophages. There have been studies of epithelial cells and invasion phenotypes associated with MAP infection. MAP-infected bovine mammary epithelial cells (MAC-T) showed a more invasive phenotype in secondary infection^26^. Everman and colleagues, using their cell culture passage model, reported that pro-inflammatory cytokines such as IL-6, CCL5, IL-8, and IL-18 were increased and that transforming growth factor-β (TGF-β) was decreased when MAP reinfected bovine MDBK cells after passage through a macrophage phase^27^. In addition, transcriptomic analysis of MAP from epithelial cell infection identified increased expression of lipid biosynthesis and lipopeptide modification^27^. Lamont and colleagues revealed that epithelial processed MAP activated a novel iron assimilation mechanism linked to nitric oxide stress and upregulated genes involved in lipid uptake compared to a single cell type infection in their coculture model^28^. From the results, we hypothesized that epithelial processing of MAP may greatly contribute to the course of infection.

To understand the exact response of immune cells against MAP infection during the initial stage of infection, we designed an epithelial passage model to mimic the course of infection in an in vitro environment. The aim of the study was to identify pathogenic pathways by comparison of the expression of cytokines and global genes of bovine peripheral blood mononuclear cells (PBMCs) in the context of MAP infection with or without epithelial processing.

## Results

### Differentially expressed genes by RNA-seq

**Figure 1:**
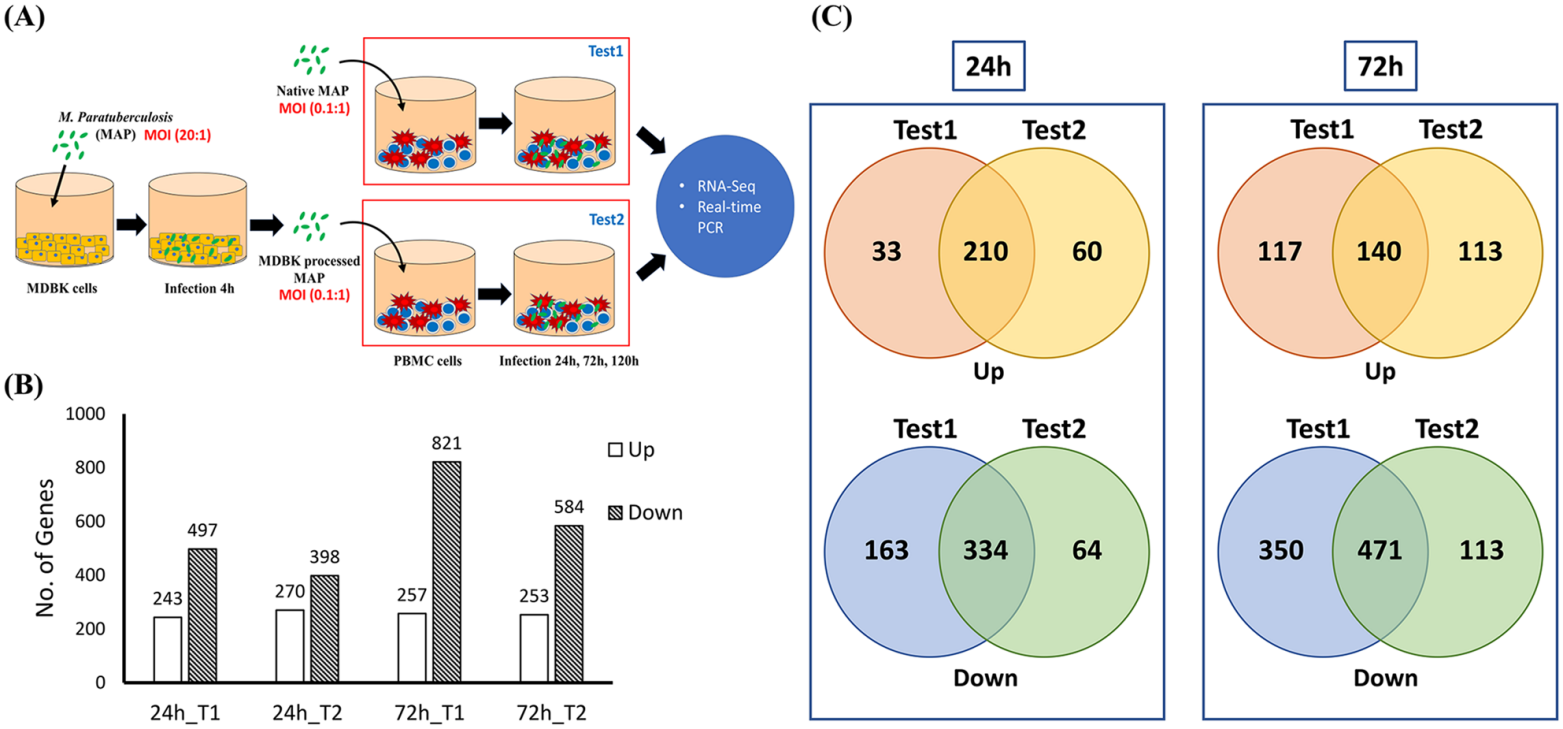
Transcriptomic profiling of bovine peripheral mononuclear cells (PBMCs). (**A**) Experimental design of the analysis. (**B**) The numbers of differentially expressed genes (DEGs). DEGs of both groups T1 and T2 were obtained when compared to non-infection control of each time point (24 h and 72 h p.i.) based on fold-change  ≥ |2.0| and p-value < 0.05. (**C**) The numbers of commonly up- and downregulated genes at 24 and 72 h post-infection.

Prior to RNA-seq, viability of MAP was checked using qRT-PCR to confirm infection to each sample. Specific amplification for sigA, the housekeeping gene of MAP, was observed in both MDBK and PBMCs at all time points, while only non-specific amplification was observed in the non-infection control (Fig. S1). Therefore, intracellular survival of MAP to both of MDBK and PBMCs was confirmed. DEG analysis was performed from RNA-seq data produced from PBMCs. DEGs were described as a fold-change value of the expression changes in each test group compared to the non-infection control for each time point, respectively. The number of DEGs in each test group is shown in Fig. 1B. In all test groups, more genes were downregulated in comparison with upregulated genes, and downregulated genes in both groups T1 and T2 was more than two times of the upregulated genes at 72 h p.i. (Fig. 1B). At the 24-h time point, number of commonly upregulated genes were higher than those of group-specific genes, while the numbers of group-specific genes and commonly upregulated genes similar at 72 h p.i. (Fig. 1C). Among the downregulated genes, the number of genes specific to group T1 was more than that specific to group T2, and the number of genes commonly downregulated was higher than the sum of T1 and T2 (Fig. 1C). As a result, it is thought that the difference of gene expression pattern increases over time between the two test groups.

### Canonical pathway analysis

**Figure 2:**
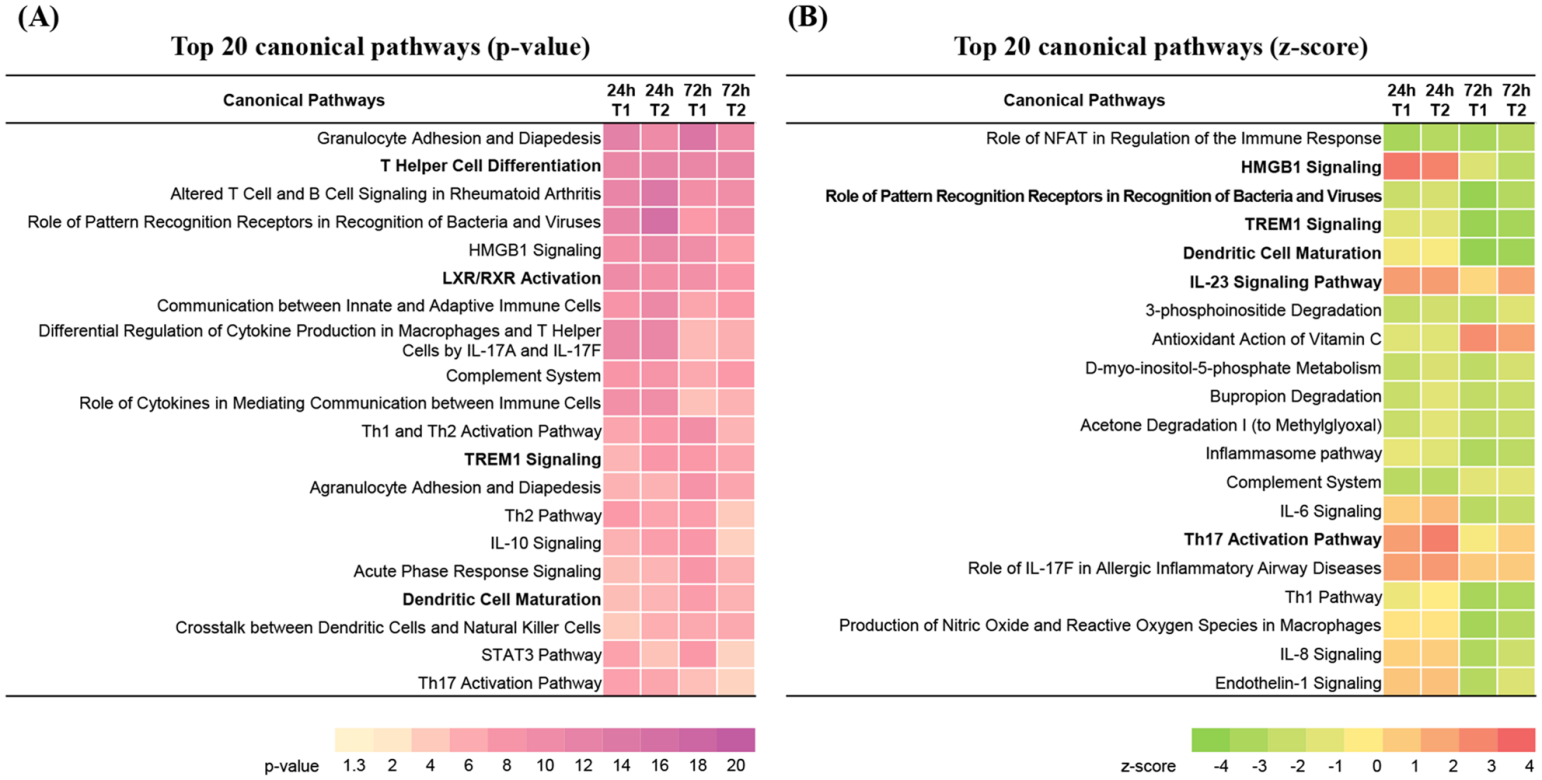
Canonical pathway analysis of DEGs in each experimental group using the IPA tool. (**A**) Top 20 canonical pathways sorted by p-value. (**B**) Top 20 canonical pathways sorted by z-score.

A canonical pathway analysis for each group was performed using the IPA tool. The top 20 canonical pathways were sorted through comparison analysis (Fig. 2). The most significant pathways according to p-value were “Granulocyte Adhesion and Diapedesis”, “T helper cell differentiation”, “Altered T cell and B cell signaling in Rheumatoid Arthritis”, and “Role of pattern recognition receptors in the recognition of bacteria and viruses” (Fig. 2A). The top 20 canonical pathways according to z-score (a score of the predicted direction of the pathway) showed a trend to overall suppression of corresponding pathways in both groups T1 and T2 at 72 h p.i., while “High mobility group box 1 (HMGB1) signaling” was found to be activated at 24 h p.i. and suppressed at 72 h p.i. in both groups (Fig. 2B). In addition, pathways such as “Th17 activation pathway” and “Role of IL-17F in allergic inflammatory airway diseases” tended to be activated at all time points in both groups. The “Role of pattern recognition receptors in recognition of bacteria and viruses”, “Triggering receptor expressed on myeloid cells 1 (TREM1) signaling” and “Dendritic cell maturation” pathways tended to be suppressed in both groups at 72 h p.i., of which the two preceding pathways showed high p-values. Therefore, these changes were thought to be among the most significant in PBMCs following MAP infection.

### Analysis of T helper cell differentiation patterns of MAP-infected PBMCs

**Figure 3:**
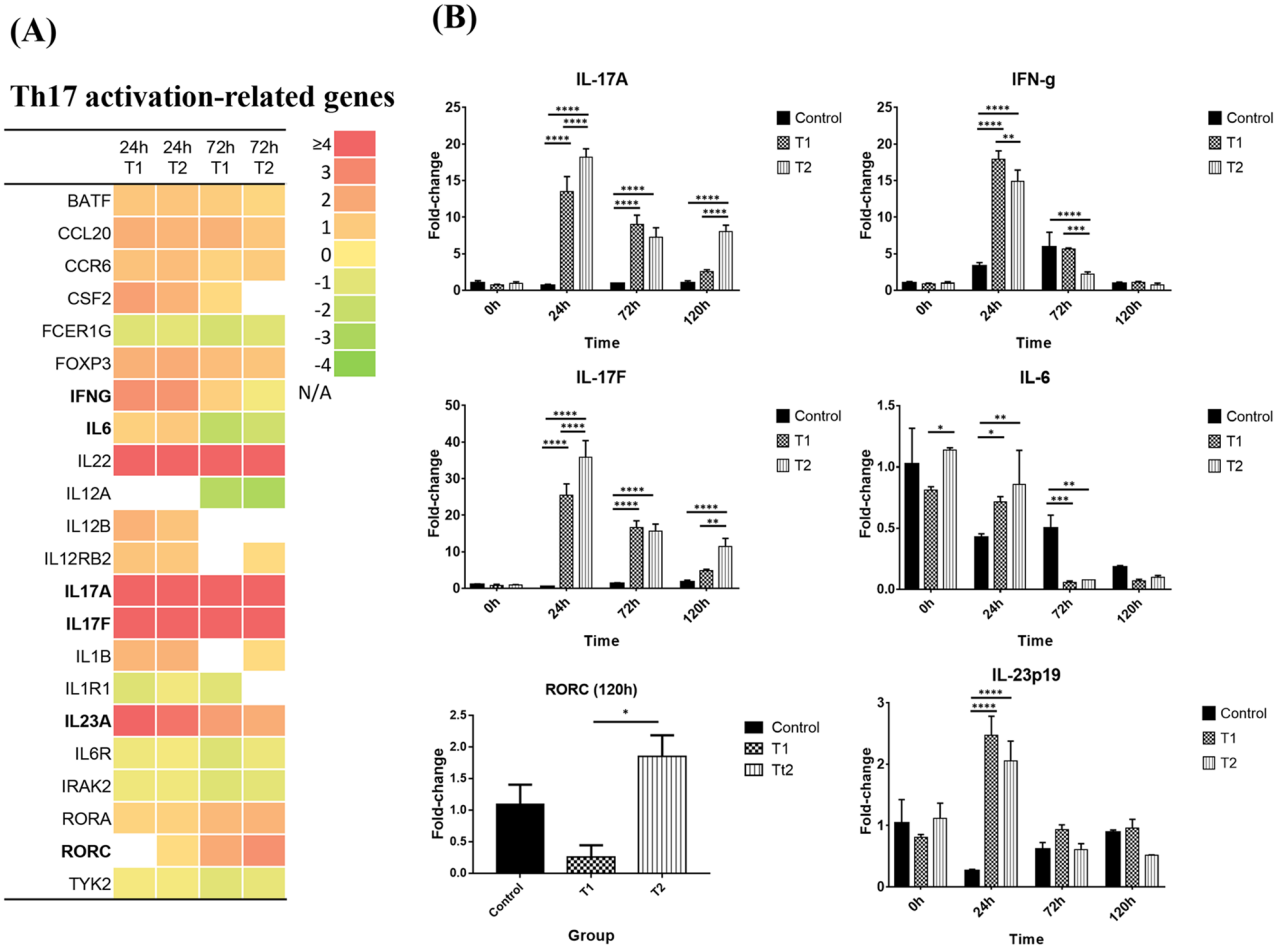
Gene expression profile associated with the Th 17 activation pathway. (**A**) Gene expression profile associated with the Th17 activation pathway annotated by the IPA tool from RNA-seq data. The log_2_ Fold-change of each group is described by a color scale. Genes that were not significant (p-value ≥ 0.05 or Log_2_FC < 1.0) are shown as N/A. (**B**) Quantitative PCR (qPCR) analysis of genes associated with Th17 differentiation. Fold-changes of each sample were calculated by 2 − ΔΔCT analysis. Statistical significance was calculated by ANOVA with Tukey’s multiple comparisons test (p-value, *< 0.05; **< 0.01; ***< 0.0005; ****< 0.0001).

As shown in Fig. 3A, one of the major immune responses of PBMCs to MAP infection was a change associated with T helper cell differentiation. The gene expression change of each group annotated to the “T helper cell differentiation” pathway is described in Fig. S2. As shown in the z-score analysis (Fig. 2B), differences in gene expression patterns of T helper cell differentiation were greater in time-dependent ways than with epithelial processing (Fig. S2). Th1-related genes were shown to be upregulated at 24 h p.i. and downregulated at 72 h p.i. in both groups. There were no significant differences in gene expression associated with Th2 differentiation compared to control samples over time. Interestingly, Treg cell differentiation-related genes were upregulated, and the increases in the expression of genes such as IL-10 or TGF-β, which are effector cytokines of Treg cells, were not observed.

**Figure 4:**
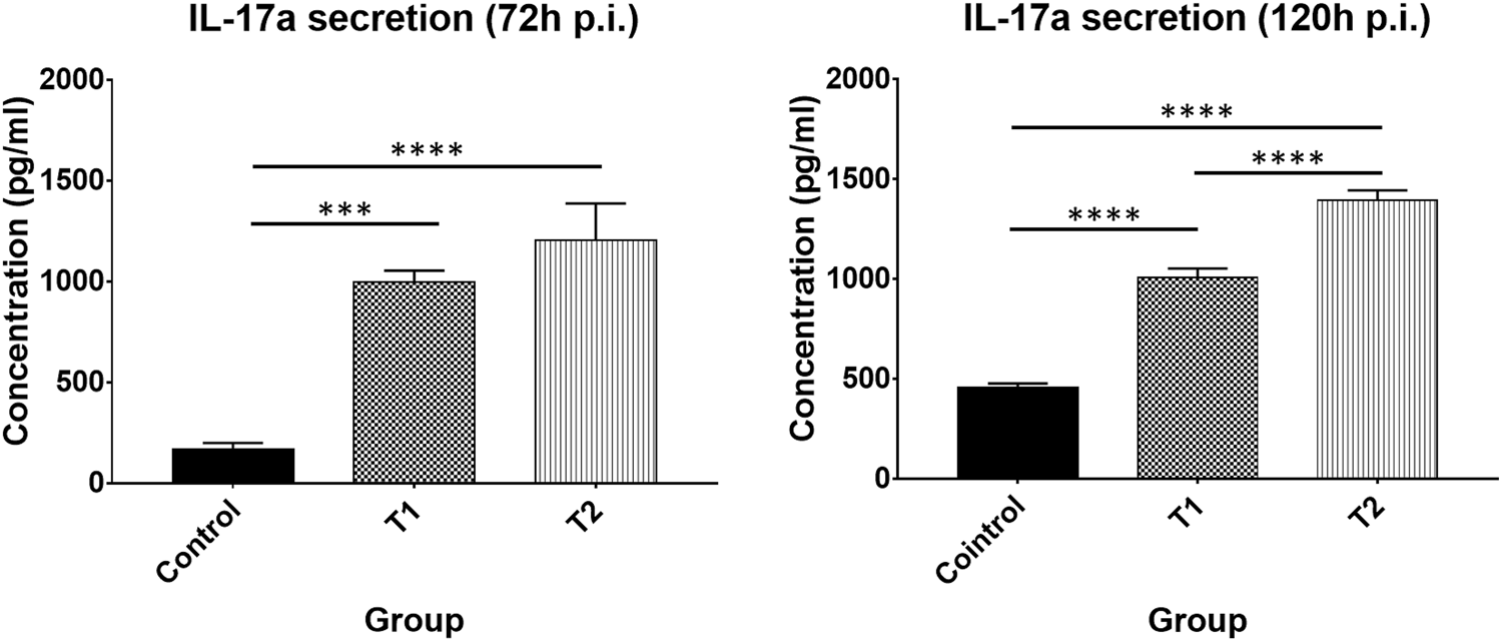
Production of IL-17a from bovine peripheral blood mononuclear cells after treatment with MAP. IL-17a in the culture supernatants of PBMCs were quantified with ELISA after infection with MDBK-processed and native MAP. Concentration of IL-17a was measured by absorbance at 450 nm based on standard curve analysis. Statistical significance was calculated by ANOVA with Tukey’s multiple comparisons test (p-value, *< 0.05; **< 0.01; ***< 0.0005; ****< 0.0001).

The most significant immunological response to MAP infection in PBMCs was considered to be the Th17-like immune response because the two cytokine genes, IL-17A and IL-17F, are most upregulated among T cell-related genes in both groups (Fig. 3A). IL-22, an effector cytokine associated with IL-17 family genes, was also highly upregulated throughout the analyzed period. Interestingly, cytokine genes associated with Th17 activation did not show differences in expression compared with the control until 72 h p.i. (IL-1B, IL-23A, IFNG) or were even downregulated (IL-6), although IL-17A and IL-17F maintained higher expression levels. CCR6 and CCL20 were slightly upregulated at both 24 and 72 h p.i. However, the RORA and RORC genes showed little difference in expression from the control at 24 h p.i. and were then upregulated at 72 h p.i., with increased expression in group T2 compared with group T1. In particular, RORC was upregulated in group T2 at 120 h, whereas T1 was downregulated. In addition, the expression levels of IL-17A and IL-17F were significantly upregulated in group T2 at 120 h p.i. compared with group T1 by real-time PCR analysis (Fig. 3B). The expression levels of IL-17A and IL-17F tended to decrease over time in both groups but were maintained up to 120 h in T2. The difference of gene expression of IL-17A was confirmed by measuring IL-17a production with ELISA in the culture supernatants. The analysis showed that secretion of IL-17a was significantly higher in group T2 than group T1 at 120 h p.i. even though concentration of IL-17a in both test groups was significantly higher than non-infection control at 72 h and 120 h p.i. (Fig. 4). Overall, it was hypothesized that initial activation of Th17-derived cytokines is the major immune response to MAP, but epithelial processed MAP induces prolonged Th17 activation.

### Downregulation of cell surface receptors

**Figure 5:**
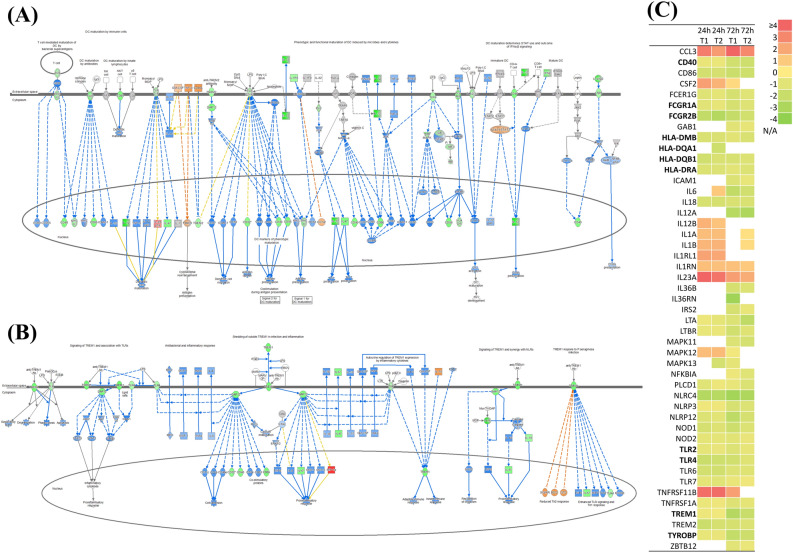
Ingenuity pathway analyses of the dendritic cell maturation pathway (**A**) and TREM-1 signaling pathway (**B**) at 72 h post-infection, and expression level of related genes (**C**). Genes shown in red indicate upregulation, genes shown in green indicate downregulation, genes shown in orange indicate predicted activation, genes shown in blue indicate predicted inhibition, and an uncolored node indicates that the genes were not differentially expressed in this pathway. The log2 Fold-change of each group is described by a color scale. Genes that were not significant (p-value ≥ 0.05 or Log_2_FC < 1.0) are shown as N/A.

Another major immunological change in PBMCs for MAP infection was downregulation of macrophage or dendritic cell surface receptor genes. Common to 72 h p.i. in groups T1 and T2, canonical pathways such as “TREM-1 signaling pathway” and “Dendritic cell maturation” showed negative z-scores. It was presumed that the reason two different canonical pathways were predicted to be suppressed is that those pathways commonly contain many surface receptor genes in phagocytic cells. Gene expression pattern of these pathway related genes were shown in Fig. 5. Downregulation of surface receptor genes related to “Dendritic cell maturation pathway” such as MHC class II, TLR2, TLR4, CD40, FcγR, and TREM2 was observed in both group T1 and T2 especially in 72 h p.i (Fig. 5A,C). It was also observed that the expression levels of surface receptor genes, such as TREM1, DAP12, and TLR4, associated with “TREM-1 signaling pathways”, were downregulated (Fig. 5B,C). The downstream function of these pathway was also predicted to be downregulated as a result of a cascade response (Fig. 5A,B). Consequently, once MAP has invaded the host cell, the host cell seems to suppress the expression of surface receptors such as TLRs and MHC class II molecules required for pathogen recognition.

### Manipulation of cholesterol metabolism by MAP

**Figure 6:**
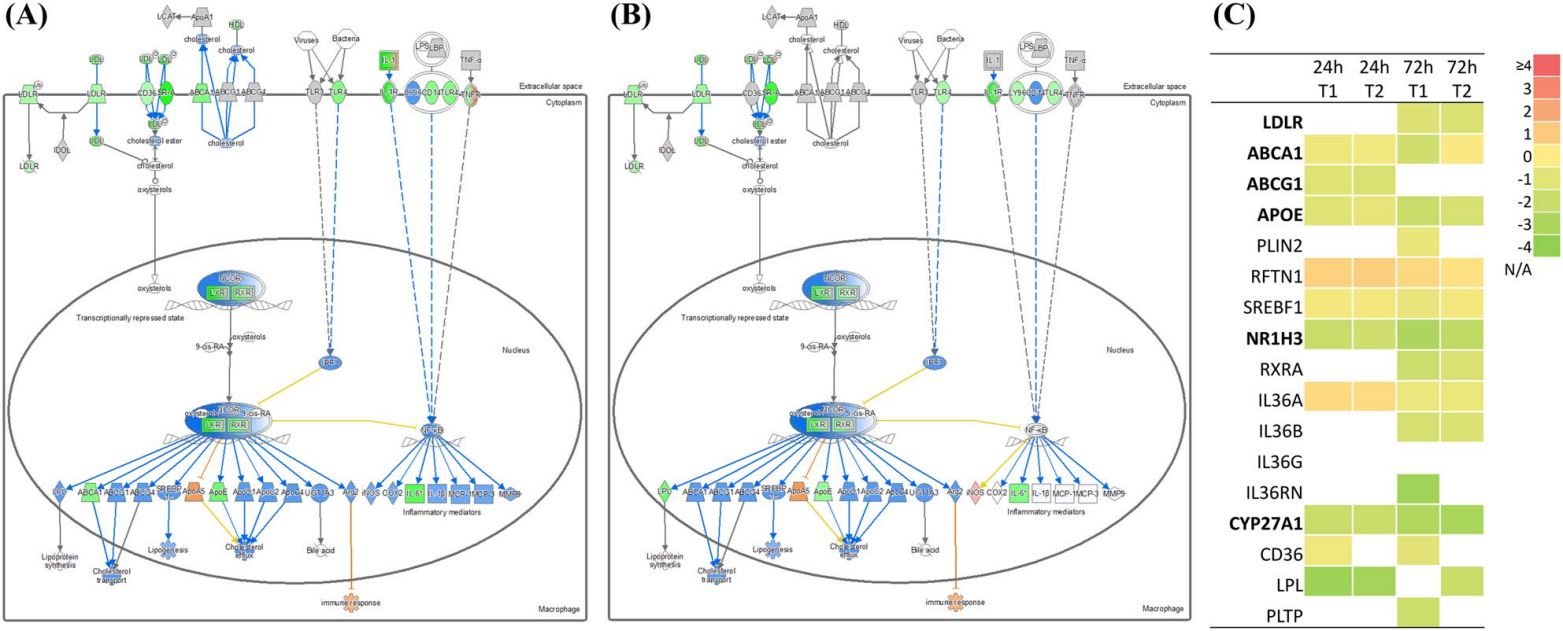
Ingenuity pathway analyses of the Liver X receptor/Retinoid X receptor pathway (LXR/RXR pathway) at 72 h post-infection in (**A**) group T1 and (**B**) group T2. Genes shown in red indicate upregulation, genes shown in green indicate downregulation, genes shown in orange indicate predicted activation, genes shown in blue indicate predicted inhibition, and an uncolored node indicates that the genes were not differentially expressed in this pathway. (**C**) Gene expression profile associated with cholesterol transport. The log2 Fold-change of each group is described by a color scale. Genes that were not significant (p-value ≥ 0.05 or Log_2_FC < 1.0) are shown as N/A.

One of the major changes of interest after infection was the “Liver X receptor/Retinoid X receptor (LXR/RXR) pathway”. The LXR/RXR pathways tended to be downregulated in both groups T1 and T2 (Fig. 6A,B). The cholesterol efflux transporters ABCA1, ABCG1, and APOE, which are regulated by the LXR/RXR pathway, were downregulated (Fig. 6C). In addition, several genes related to cholesterol metabolism were also downregulated, such as CD36, CYP27A1 and LDLR.

Additional analysis with DEGs (fold-change ≥|1.5| and p-value < 0.05) between groups T1 and T2 using the IPA tool showed that the key pathway was the “LXR/RXR activation pathway”, which affects cholesterol biosynthesis and other pathways (Fig. S3). Compared to group T1 at 72 h p.i., group T2 showed downregulation of genes related to “cholesterol biosynthesis” and upregulation of genes related to “LXR/RXR activation pathway” (data not shown). Although the LXR/RXR activation pathway was downregulated in both groups compared to the control, it was presumed that group T2 had a more protective response than group T1 (or that group T1 showed a more pathogenic response than group T2).

## Discussion

The role of epithelial cells as a first-line defender in infection with MAP has been studied by many researchers^26–28^. Janagama and colleagues reported that MAP transcriptional profiles isolated and enriched from the ileum and mesenteric lymph nodes from naturally infected cattle are significantly divergent from straight macrophage infection^29^. The possible reasons for the difference between in vivo and in vitro findings have been described as epithelial processing of MAP and crosstalk between epithelium and macrophages^28^. Previously, it was reported that the influence of epithelial processing in MAP infection was because MAP infecting epithelial cells showed a more invasive phenotype when reinfecting other epithelial cells^26,27^.

In the present study, global gene expression patterns in PBMCs that were infected with MAP with or without epithelial processing were analyzed. The major difference between the two groups was that PBMCs infected with MDBK-processed MAP showed prolonged upregulation of IL-17A and IL-17F in entire time points (until 120 h p.i.), while PBMCs infected directly with MAP showed continuous decrease of corresponding genes despite significant initial upregulation (24 h p.i.).

IL-17 is one of the major expressed cytokines in chronic infections such as mycobacterial infection or autoimmune diseases such as Crohn's disease, psoriasis, and rheumatoid arthritis^30–34^. Secretion of IL-17a in the early phase of microbial infection was considered as an innate nonspecific immune response that enhances neutrophil chemotaxis by promoting IL-6, granulocyte colony-stimulating factor (G-CSF) and CXCL8 (also known as IL-8) production^35,36^. Naïve CD4+ T cells are differentiated into specific lineage effector T helper cells by binding to the MHC class II/antigen complex labeled from antigen presenting cells (APCs) and interacting with costimulatory cytokines such as TGF-β, IL-6, IL-21, IL-23 and IL-1β^35^. In vitro differentiation of bovine Th17 cells was induced by IL-6 and TGF-β^37^. Initial activation of naïve CD4+ T cells with these specified polarizing factors results in upregulation of signal transducer and the activator of transcription 3 (Stat3) and retinoic acid receptor-related orphan receptor-γt (RORγt) to enhance IL-23 responsiveness and induce IL-17 production.

Our results showed relative upregulation of RORC and IL-21 in the T2 group compared with the T1 group at 72 h p.i., and even the gene expression level of RORC in the T1 group was downregulated at 120 h p.i., while the increase in T2 was maintained. Furthermore, Th17-activation-related canonical pathways (Th17 activation pathway and IL-23 signaling pathway) in group T2 showed higher z-scores than in group T1 (Fig. 2B). However, the differential expression of TGF-β was not observed at 24 and 72 h p.i., and IL-6 and IL-6R were downregulated at 72 h p.i. Because these polarizing cytokines are produced at the initial stage of infection and the differentiation process of specific T helper cells takes approximately 3 days in vitro^37^, further analysis with DEGs at earlier time points (ex. 4–8 h p.i.) is needed to confirm activation and stimulation of Th17 differentiation.

The best studied role of IL-17 in mycobacterial infection is related to granuloma formation^32,38^. In addition to TNF-α or IFN-γ, IL-17a contributes to the formation of granulomas against mycobacterial infection^32^. Gopal and colleagues suggested that IL-17 played a role in granuloma maturation in *M. bovis* BCG strain infection^39^. The authors found that IL-17A−/− mice showed impairments in developing mature granulomas 28 days after BCG infection in the lung. Our previous study suggested that suppression of the Th17-derived immune response in cattle infected with MAP resulted in loss of granuloma and enhanced survival of MAP during subclinical stages^20^.

From the differences in expression of IL-17 genes by the epithelial processing and the importance of IL-17 cytokine in mycobacterial infection, we hypothesized that epithelial processing causes Th17 inducible response against MAP infection. A previous study associated with epithelial processing, Lamont and colleagues showed that intracellular MAP infected to MAC-T cell changes its gene expression related to mycolic acid building and DNA repair^28^. Another mycobacterial study with *M. smegmatis* (MSM) suggested that epithelial cell exposed bacteria had significant increase in intracellular growth during secondary infection with human macrophages^40^. Epithelial processed MSM induced cytokines such as IL-1β, IL-6, IL-8, IL-12, TNF-α, MIP-1α, and MCP-1 in THP-1 cell compared to native MSM at 24 h p.i.^40^. Although there is no direct link between the epithelial processing and Th17 differentiation, cell wall rebuilding and changes of infection phenotypes after epithelial infection may contribute to change of antigen recognition and presentation in phagocytic cells. Further analysis is needed to confirm the effect of epithelial processing on MAP.

The second characteristic observed was that global gene suppression was common at 72 h p.i. In particular, cell surface receptor genes were commonly downregulated at 72 h p.i. The negative z-scores in “TREM1 signaling pathway” and “Dendritic cell maturation pathway” showed that surface receptor genes such as TLRs, NLRs, TREM and FcγR are commonly downregulated and affected downstream suppression (CD40, CD86, MHC class molecules). Downregulation of the overall phagocytic cell surface receptor gene was also observed in the Mtb infection model for human whole blood^41^. The authors suggested that when mycobacteria enter a phagocytic cell, the key pathway related to antigen recognition, intracellular killing and antigen presentation to T or B cells is suppressed by downregulation of cell surface receptor genes. The overall downregulation of genes was also observed in bovine MDMs against MAP strain L1 infection after 24 h by RNA-seq^25^. The Mtb glycoprotein Rv1016c showed inhibition of DC maturation and impaired Th1/Th17 responses in a mouse model^42^. Our findings suggest that downregulation of IL-1B, IL-6, IL-12, IL-18 and IFNG affected by impairment of phagocytic cells in 72 h p.i. might result in suppression of Th1 cell activation.

The last observed characteristic phenomenon was suppression of the LXR/RXR pathway. Pathogenic mycobacteria are known to alter the lipid metabolism of the host to survive in the intracellular environment^43,44^. In particular, carbon source is obtained by metabolizing cholesterol among host-derived lipids^45^. Changes in the expression of cholesterol and lipid metabolism-related genes were observed in the early stage infection of cattle infected with MAP^46^. Cholesterol has also been reported to be an essential factor in the phagocytosis of macrophages and the inhibition of phagocytosis^47^. In this study, it was observed that LXR/RXR, which regulates gene expression related to cholesterol transport in the host cell, was downregulated in both groups at 72 h p.i. The ABCA1, ABCG1 and APOE that are regulated by the LXR/RXR pathway are involved in cholesterol efflux^48^. A similar observation has been reported by Johansen and colleagues^49^. The authors showed that murine macrophages (RAW264.7) infected with MAP resulted in accumulation of cholesterol at 72 h p.i. and also showed downregulated expression of APOE, which was regulated by the LXR/RXR pathway. However, activation of the LXR/RXR pathway against Mtb infection induced a protective immune response by growth restriction^50^. Our previous study showed activation of the LXR/RXR pathway in whole blood naturally infected with MAP in a later stage of infection^51^. Downregulation of the LXR/RXR pathway is thought to be a pathogenic mechanism of MAP at the initial stage of infection. An important role of LXR is suppression of cholesterol biosynthesis^52^. Cholesterol homeostasis is regulated by intracellular levels of oxysterols through the activation of LXR^53^. Our results showed that an enzyme that converts cholesterol to oxysterol, CYP27A1, was downregulated at 72 h p.i., which might have resulted in downregulation of LXR.

In summary, the results from this study suggest that two distinctive molecular mechanisms were actively working against MAP infection. One is that epithelial processing of MAP might induce prolonged Th17 activation in PBMCs. It was supported by the prolonged gene expression of IL-17A/F and RORC at 120 h p.i. and significantly higher concentration of IL-17a in the group T2. Another is that MAP (w/wo epithelial processing) survives in immune cells by modulating host lipid metabolism and switching off intracellular killing pathways after 72 h of infection. It was supported by the observation of downregulation of genes involved in lipid metabolism, antigen recognition, processing and presentation at 72 h p.i.. In terms of host–pathogen interaction, epithelial processing would be a pathogenic mechanism for long term survival of MAP or the host’s protective response to avoid immunopathology such as excessive inflammation by activation of Th17 response and suppression of Th1 response. To clarify this, further studies are needed from the perspective of bacteria. One of the key points is to show how epithelial processing affect MAP phenotype in terms of antigenic modification.

## Materials and methods

### Ethics statement

This study was conducted in an approved facility in strict accordance with all university and federal regulations. Animal experiments were performed according to the principles established by the Animal Protection Act and the Laboratory Animal Act in the Republic of Korea. All experiments were reviewed and approved by the Institutional Biosafety Committee (Approval no. SNUIBC-R190109-1) and the Institutional Animal Care and Use Committee at Seoul National University (Approval no. SNU-190124-4).

### Preparation of bacteria

The MAP ATCC19698 strain was grown at 37 °C in Middlebrook 7H9 broth (Beckton Dickinson) supplemented with Mycobactin J (2 mg/L, Allied Monitor, Fayette, MO), 0.04% Casitone, 0.2% glycerol and 10% oleic acid-albumin-dextrose-catalase enrichment (OADC) for 3–4 weeks. When the optical density at 600 nm (OD_600_) reached 1.0 (considered to be 10^8^ cfu/mL), the bacterial suspension was divided into 1-mL aliquots and pelleted. Bacterial pellets were stored at − 80 °C after the removal of media.

### Mammalian cell culture

Madin–Darby bovine kidney (MDBK) epithelial cells were cultured in Dulbecco's Modified Eagle's Medium (DMEM) supplemented with 10% heat-inactivated FBS (Gibco) at 37 °C in 5% CO_2_. Bovine PBMC cells were isolated from a JD-negative Holstein cow. The selected cow was housed on an experimental farm of Seoul National University, and all animals were negative to JD continuously using commercial ELISA kit (IDEXX Laboratories, Inc., Westbrook, ME, USA). Whole blood was collected in T-75 flasks containing 100 mL of RPMI 1640 media supplemented with 10% fetal bovine serum (FBS), 1% penicillin/streptomycin and heparin (2000 units/mL). 500 mL of blood was collected in five T-75 flasks and immediately transferred to the laboratory. Whole blood was then centrifuged (900×*g*, 4 °C for 20 min) for isolation of buffy coat. Collected white blood cells from buffy coat were diluted with RPMI 1640 media and layered on Histopaque 1.077 g/mL (Sigma Aldrich, Taufkirchen, Germany). PBMCs were isolated via density gradient centrifugation (400×*g* for 30 min, RT, deceleration: 0). Isolated PBMCs were washed twice and resuspended in RPMI 1640 containing 20% FBS and 1% penicillin/streptomycin and then cultured for 24 h at 37 °C.

### Epithelial passage model

To analyze how the epithelial processing of MAP influences immune cells, an epithelial passage model was described with modifications from Everman et al. (Fig. 1A). Bovine MDBK cells (5 × 10^5^ cells/well) were infected with MAP ATCC19698 for 4 h (MOI 20:1). Live MAPs passed through MDBK for 4 h were isolated by differential centrifugation, as previously described^27,54^). MDBK cells infected with MAP were washed twice with DPBS and lysed by 0.1% Triton X-100 solution for 15 min. The lysate was then pelleted by centrifugation for 15 min (2000×*g*, 4 °C) and resuspended 0.1% Triton X-100 again. Intact MDBK cells and debris were eliminated by pelleting by centrifugation at 60×*g* for 3 min. Suspension containing bacteria was centrifuged again at 2000×*g* for 15 min, and the pellet was resuspended in RPMI 1640 media. Residual debris was eliminated by centrifugation at 60×*g* for 3 min again, and bacteria were centrifuged at 5600×*g*, 4 °C for further use. Bovine PBMCs (5 × 10^6^ cells/well) were infected with native MAP (group T1) and MDBK-processed MAP (group T2) for 24, 72, and 120 h (MOI 0.1:1).

### RNA extraction

Total RNA from MDBK and PBMCs was extracted using RNeasy Mini Kit (Qiagen, Germany) following the manufacturer’s instruction with some modification. Between the addition of buffer RLT and addition of 100% EtOH, a bead beating step was added to efficiently destroy the MAP cell wall. Briefly, lysis buffer (RLT) was added to cells and subjected to 2 mL screwcap tube containing 0.1 mm silica/zirconia beads. Then, bead beating was performed two times using FastPrep-24 5G instrument (MP biomedicals, CA, USA) for 45 s at speed setting of 6.5. Lysate was centrifuged at 13,000×*g* for 2 min, and supernatant was subjected to next step. Purity of all extracted RNA was checked to measure A260/230 and A260/280 value using Nanodrop ND-1000 instrument (Thermo Fisher Scientific, MA, USA).

### RNA-seq analysis

Among the PBMC samples of three time points, the samples at 24 h and 72 h p.i. were selected for RNA-seq analysis. Subsequent RNA preparation steps were carried out at the TheragenEtex Bio Institute (Seoul, Korea). The purity and integrity of RNA were determined using an Agilent 2100 Bioanalyzer (Agilent Technologies, CA, USA), and the samples that had a RIN greater than 7.0 were used for RNA library construction. The samples were then used to generate sequencing libraries with a TruSeq Stranded mRNA Sample Preparation Kit (Illumina, CA, USA) and were sequenced on an Illumina NovaSeq 6000 sequencer following the manufacturer’s instructions. After filtration of low-quality reads, mapping of the reads to the reference genome related to the species was performed using the TopHat aligner^55^. The gene expression level was measured with Cufflinks v2.1.1^56^ using the gene annotation database of the species. To improve the accuracy of the measurement, multiread-correction and frag-bias-correction options were applied. All other options were set to default values.

### Transcriptomic analysis

Differentially expressed genes (DEGs) of both groups T1 and T2 were obtained when compared to non-infection control of each time point (24 h and 72 h p.i.) based on fold-change ≥|2.0| and p-value < 0.05. The canonical pathways and functional analyses of the DEGs were generated through the use of IPA, a software that enables analysis, integration, and understanding of data from global gene expression using knowledge-based database (QIAGEN Inc., https://www.qiagenbioinformatics.com/products/ingenuity-pathway-analysis)^57^.

### Quantitative PCR analysis

Cytokine genes related to Th17 activation (IL-17A, IL-17F, IL-23p19, IFNG, IL-6 and RORC) were subjected to quantitative PCR (qRT-PCR) analysis with RNA samples at 24, 72, and 120 h p.i. to verify RNA-seq analysis data and to check the tendency of the immune response at 120 h p.i. In addition, viability of intracellular MAP (infected to MDBK and PBMCs) was checked by qRT-PCR with sigA gene which is housekeeping gene of MAP. Briefly, reverse transcription of mRNA to cDNA was carried out using a Quantitect Nova Reverse Transcription Kit (Qiagen, Germany). The primers used are listed in Table S1. Real-time PCR was performed with 2 μL of cDNA using the Rotor-Gene SYBR Green PCR Kit (Qiagen) and a Rotor-Gene Q real-time PCR cycler (Qiagen) with the following PCR conditions: 95 °C for 5 min for initial denaturation followed by 45 cycles at 95 °C for 15 s and 60 °C for 45 s. The GAPDH was used as an internal control. Relative expression was calculated using the comparative CT (2−Δ ΔCT) method.

### ELISA

Secreted level of IL-17a in culture supernatants was assayed with commercially available ELISA kit (Raybiotech, GA, USA). All procedures were conducted according to the manufacturer's instructions. The results were determined by measuring the absorbance at 450 nm. Concentration of each sample was calculated based on standard curve analysis.

### Statistical analysis

Statistical significance of qPCR and ELISA results was confirmed by ANOVA with Tukey’s multiple comparisons test among the experimental groups using GraphPad Prism software version 7.00 (GraphPad Software, Inc., La Jolla, CA, USA). When the p-value of each test was less than 0.05, it was considered statistically significant. All experiments were recorded as the means of biological triplicates.

## Supplementary information

Supplementary Information.

## Data Availability

All datasets used in the RNA-seq transcriptomic analysis are available at Gene Expression Omnibus (GEO) under accession number GSE149494 (https://www.ncbi.nlm.nih.gov/geo/query/acc.cgi?acc=GSE149494).
