# Supplementary material for: Epithelial processed Mycobacterium avium subsp. paratuberculosis induced prolonged Th17 response and suppression of phagocytic maturation in bovine peripheral blood mononuclear cells
Source: Sci Rep. 2020 Dec 3;10:21048. doi: 10.1038/s41598-020-78113-8 (PMC7713309; doi:10.1038/s41598-020-78113-8)
Supplement: Supplementary file 1 — Supplementary Information. [file 41598_2020_78113_MOESM1_ESM.pdf]

Epithelial processed *Mycobacterium avium* subsp. *paratuberculosis* induced prolonged Th17 response and suppression of phagocytic maturation in bovine peripheral blood mononuclear cells.

Hong-Tae Park[1], Hyun-Eui Park[2], Soojin Shim[1], Suji Kim[1], Min-Kyoung Shin[2], and Han Sang Yoo[1,3]*

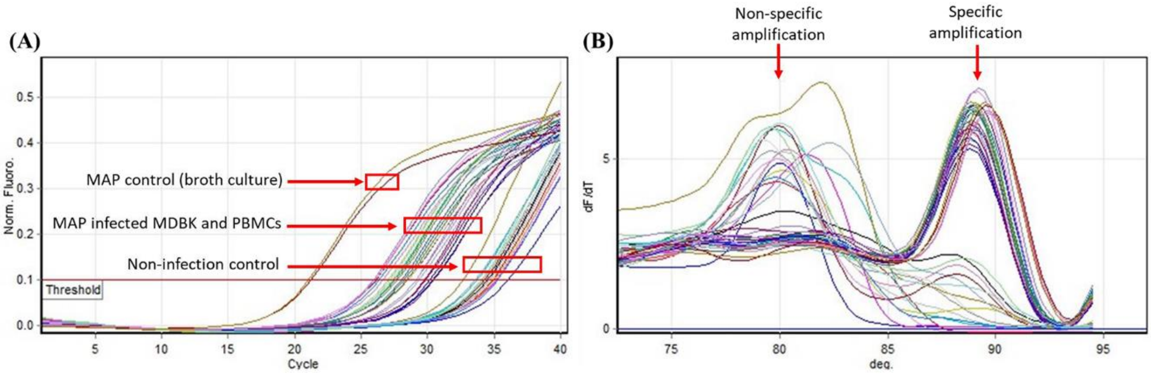

Supplementary Figure S1. Amplification of sigA gene from total RNA by real-time PCR analysis. Real-time PCR based on SYBR green method was conducted with total RNA from MAP-infected MDBK and PBMC samples. (A) Quantitative analysis plot. Non-infection control samples showed lower Ct values due to the non-specific amplification. (B) Melt curve analysis. All MAP infected samples including positive control showed single peak which indicated specific amplification.

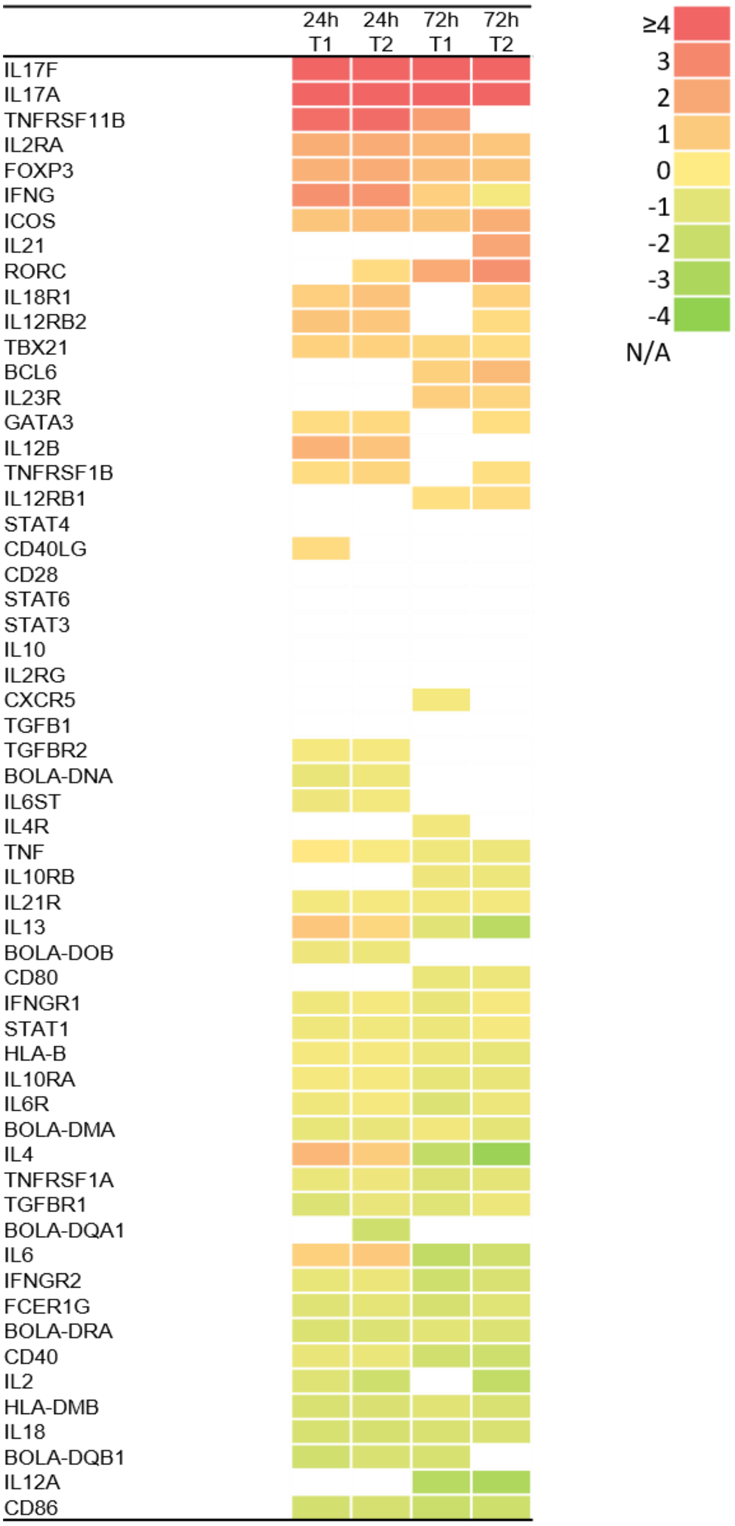

Supplementary Figure S2. Gene expression profile associated with T helper cell differentiation. Listed genes were annotated by the IPA tool. The log2 Fold-change of each group is described by a color scale. Genes that were not significant (p-value ≥ 0.05 or $Log_2FC<1.0$) are shown as N/A.

PBMC_72h_T1_T2_IPA_0.05_0.6 - Overlapping Canonical Pathways

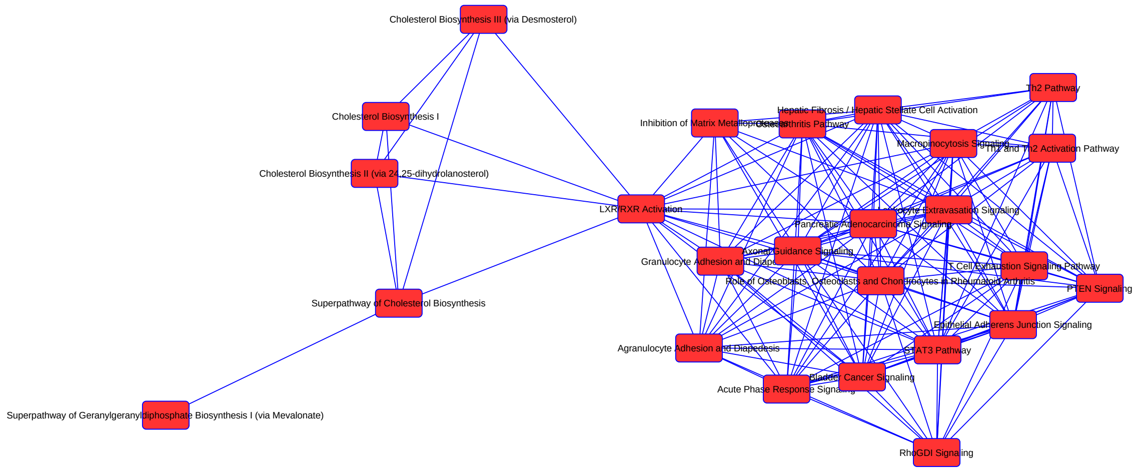

© 2000-2019 QIAGEN. All rights reserved.

Supplementary Figure S3. Overlapping canonical pathways between group T1 and T2 analyzed by the IPA tool. DEGs directly compared between groups T1 and T2 at 72 h post-infection (p-value<0.05 and Fold-change≥1.5) were used in the analysis. The LXR/RXR activation pathway was the key pathway that enabled relative activation of cholesterol efflux and suppression of cholesterol biosynthesis of group T2 compared to T1.

Supplementary Table S1. List of quantitative real-time PCR primers used in this study.

Target Gene		Oligonucleotide sequence (5'→3')	Product size (bp)	Accession No.
GAPDH	F	GGCGTGAACCACGAGAAGTATAA	120	NM_001034034.2
	R	CCCTCCACGATGCCAAAGT		
IL-17A	F	CACAGCATGTGAGGGTCAAC	101	NM_001008412.2
	R	GTGGAGAGTCCAAGGTGAGG		
IL-17F	F	GAGGAAGCAAAACGGCTGTC	115	NM_001192082.1
	R	CTGATCTGCCATCGGGTCAT		
IFN-γ	F	CAAATTCCGGTGGATGATCTGC	157	NM_174086.1
	R	GGCAGGAGGACCATTACGTT		
IL-6	F	GGGCTCCCATGATTGTGGTA	69	NM_173923.2
	R	GTGTGCCCAGTGGACAGGTT		
IL-23p19	F	CAACAGCTCTCACAGCAACTC	178	NM_001205688.1
	R	TTTGCAAGCAGGACTGACTGTT		
RORc	F	GGGCAGGGAGAACTTCTATG	135	NM_001083451.2
	R	TCTGCACCAGGTGCTCAATC		
sigA	F	CCATCTGCTGGAAGCGAATC	152	NC_002944.2
	R	AGAACTTGTAGCCCTTGGTGTAGTC		3148633-3150162 (+)
